# Review of Genetic Variation as a Predictive Biomarker for Chronic Graft-Versus-Host-Disease After Allogeneic Stem Cell Transplantation

**DOI:** 10.3389/fimmu.2020.575492

**Published:** 2020-10-19

**Authors:** Jukka Partanen, Kati Hyvärinen, Heike Bickeböller, Katarzyna Bogunia-Kubik, Rachel E. Crossland, Milena Ivanova, Francesca Perutelli, Ralf Dressel

**Affiliations:** ^1^ Finnish Red Cross Blood Service, Research and Development, Helsinki, Finland; ^2^ Department of Genetic Epidemiology, University Medical Center Göttingen, Göttingen, Germany; ^3^ Hirszfeld Institute of Immunology and Experimental Therapy, Polish Academy of Sciences, Wroclaw, Poland; ^4^ Haematological Sciences, Translational and Clinical Research Institute, Newcastle University, Newcastle upon Tyne, United Kingdom; ^5^ Medical University, University Hospital Alexandrovska, Sofia, Bulgaria; ^6^ Section of Hematology, Department of Clinical and Experimental Medicine, University of Pisa, Pisa, Italy; ^7^ Institute of Cellular and Molecular Immunology, University Medical Center Göttingen, Göttingen, Germany

**Keywords:** graft versus host disease (GvHD), biomarkers, genetic screen, stem cell transplantation (HSCT), gene marker

## Abstract

Chronic graft-versus-host disease (cGvHD) is one of the major complications of allogeneic stem cell transplantation (HSCT). cGvHD is an autoimmune-like disorder affecting multiple organs and involves a dermatological rash, tissue inflammation and fibrosis. The incidence of cGvHD has been reported to be as high as 30% to 60% and there are currently no reliable tools for predicting the occurrence of cGvHD. There is therefore an important unmet clinical need for predictive biomarkers. The present review summarizes the state of the art for genetic variation as a predictive biomarker for cGvHD. We discuss three different modes of action for genetic variation in transplantation: genetic associations, genetic matching, and pharmacogenetics. The results indicate that currently, there are no genetic polymorphisms or genetic tools that can be reliably used as validated biomarkers for predicting cGvHD. A number of recommendations for future studies can be drawn. The majority of studies to date have been under-powered and included too few patients and genetic markers. Like in all complex multifactorial diseases, large collaborative genome-level studies are now needed to achieve reliable and unbiased results. Some of the candidate genes, in particular, *CTLA4*, *HSPE*, *IL1R1*, *CCR6*, *FGFR1OP*, and *IL10*, and some non-HLA variants in the HLA gene region have been replicated to be associated with cGvHD risk in independent studies. These associations should now be confirmed in large well-characterized cohorts with fine mapping. Some patients develop cGvHD despite very extensive immunosuppression and other treatments, indicating that the current therapeutic regimens may not always be effective enough. Hence, more studies on pharmacogenetics are also required. Moreover, all of these studies should be adjusted for diagnostic and clinical features of cGvHD. We conclude that future studies should focus on modern genome-level tools, such as machine learning, polygenic risk scores and genome-wide association study-transcription meta-analyses, instead of focusing on just single variants. The risk of cGvHD may be related to the summary level of immunogenetic differences, or whole genome histocompatibility between each donor-recipient pair. As the number of genome-wide analyses in HSCT is increasing, we are approaching an era where there will be sufficient data to incorporate these approaches in the near future.

## Introduction

Allogeneic hematopoietic stem cell transplantation (HSCT) is a potentially curative treatment for hematological malignancies; up to 400 000 HSCTs have been performed worldwide to date (1). Chronic graft-versus-host disease (cGvHD), together with the acute form of GvHD (aGvHD) and relapse, are major hurdles of successful HSCT. cGvHD is an autoimmune-like disorder affecting multiple organs and involves a rash, tissue inflammation and fibrosis. Despite many advances in HSCT, the incidence of cGvHD has been reported to be between 30-60%. Notably, cGvHD can occur in different forms that likely have important pathophysiological differences. The progressive form develops directly based on an aGvHD, the quiescent form occurs in patients after recovering from aGvHD, while the *de novo* form is a cGvHD in patients who never had an aGvHD. The symptoms in most cases present within the first year, but may occur up to 3 years after HSCT. The overall incidence of cGvHD may have even increased over time, due to more frequent use of peripheral blood as the stem cell source, or due to reduced intensity conditioning regimens.

A greater likelihood of cGvHD is observed after transplantation from a haploidentical family member, especially from a female donor to her son (2, 3). This may be due to genetic disparities between the mother and her offspring, which can lead to allosensitisation and induce an immune response through antibodies directed against paternal human leukocyte antigens (HLA) and paternal minor histocompatibility antigens (miHA) such as HY-antigens (4). On the other hand, a HLA mismatch in haploidentical settings may be beneficial and facilitate graft-versus-leukemia (GvL) effect.

GvHD, as well as the desired, curative graft-versus-leukemia (GvL) effect, results from immunogenic differences between the donor and recipient. Lack of immunological tolerance by the graft immune cells is a key element. In aGvHD immunological triggers, initiated by microbial structures released from the gut during preconditioning, is a prominent component leading to cytokine storm and activation of cell-mediated immunity against structures detected as foreign by the graft immune cells. The gut along with skin and the liver are key targets of aGvHD. In cGvHD, B cells and an autoimmune-like lack of immunological tolerance are assumed to play central roles. Diagnosis and scoring of cGvHD has been challenging, as it is a heterogeneous disease with pleiotropic symptoms and clinical definitions changed over time. However, the National Institute of Health Consensus Criteria has provided a framework for more defined classification (5).

Genetic disparities between the recipient and donor strongly influence the outcome of HSCT (6). In addition to human leukocyte antigen (HLA) genes, miHAs, and non-HLA polymorphisms may contribute to susceptibility to GvHD, relapse and survival-related outcomes (7–9). The effect of genetic components on GvHD is complex and potentially comprises numerous variants in donor and recipient genomes, related to the initiation and perpetuation of GvHD. To date, the majority of genetic variation studies in the field of GvHD involve selected candidate genes, single nucleotide variants (SNPs) and small study populations (10). The methods employed by the community have rather slowly evolved toward broader approaches, and the first genome-wide association studies (GWASs) on GvHD were published by Sato-Otsubo et al. (11) and Bari et al. (12) in 2015. The absence of variants having large effect sizes, combined with the low and unbalanced case/control numbers, introduces another challenge to overcome. The success of genetic association studies depends on adequate statistical power and detecting causal variants of complex diseases requires thousands of cases and controls (13). Currently, we are not reliably able to predict the risk of developing cGvHD. There is therefore a clear unmet clinical need for predictive biomarkers that could be used prior to HSCT for risk assessment. Biomarkers can also provide us with valuable insight into the key pathogenic pathways and molecules implicated in cGvHD. The present paper reviews the current findings of using genetic variation in the patient and donor for predicting the occurrence of cGvHD. Genetic variation can influence the risk for cGvHD in at least three mutually non-exclusive ways (**Table 1**).

**Table 1:** Three non-exclusive genetic concepts or models in genetic susceptibility to graft-versus-host disease after allogeneic stem cell transplantation.

Model	Mechanism	Examples	Study tools
**High/low responder**	Genetic variants leading to differences in strength of alloimmune response	Genetic polymorphisms in *TNF* or *IL10* genes	Case (GvHD pos) – control (GvHD neg) associations; GWAS; functional genomics; eQTL
**Matching**	Amino acid or expression differences in proteins between donor and recipients leading to immune activation	HLA; deletions; minor histocompatibility antigen; genome level matching scores	Genomic level or candidate gene differences in each transplantation case; proteogenomics
**Pharmacogenetic**	Genetic variation in genes related to the effects of the drug	SNP in *IMPDH1* gene	Association of drug levels or metabolism with genetic variation in patient and with GvHD; detailed *in vivo* measurements of drug levels

- First, the patient or donor may have genetic variants that regulate the threshold or power of the immune response against foreign molecules. An individual can be a high or low responder as a result of having particular gene variants or an overall genomic profile producing e.g. higher or lower levels of regulatory cytokines, such as interleukin (IL)-10 or tumor necrosis factor (TNF)-α.
- The second level of genetic variation is related to the level of histocompatibility or “matching” between the donor and recipient. The HLA-matching is the central component, but matching of known miHAs as well as the sum score of antigenic differences may also be related to cGvHD risk. Indeed, any amino acid difference in proteins between the donor and recipient may be recognized by the immune system as foreign, if presented by the major histocompatibility complex (MHC) class I molecules to CD8^+^ T cells, leading to killing of the cells expressing the protein. Recognizing foreign peptides presented by MHC class II molecules may also lead to the activation of CD4^+^ T cells.
- Finally, genetic variation may affect cGvHD risk by pharmacogenetic differences. It should be noted that despite all modern immunosuppression and other treatments, a substantial proportion of HSCTs still results in GvHD. Genetic variants can regulate, for example, the distribution or efficacy of immunosuppressive drugs used in HSCT.

In the present review, we systematically screened published articles related to genetic associations with cGvHD and classified them into these three genetic models (**Table 1**).

Compared to quantitative biomarkers, such as protein or mRNA levels, determination of genetic variation is technically relatively simple and reliable. As the HLA-matching is anyway performed based on DNA technology, such as sequencing, determination of additional genetic variation for more reliably predicting the outcome of HSCT could be feasible in clinical practice. However, as for any biomarker, candidate genetic markers should be validated in independent series of HSCT studies with sufficient numbers of well-characterized cases. In addition, prospective studies on their specificity and sensitivity should be carried out. Currently, there is no genetic variation, barring HLA-matching, that could fulfill the criteria for a validated biomarker, or that could be in more general clinical use to predict cGvHD, aGvHD or relapse. However, there are substantial advances taking place with candidate gene approaches, GWASs and more sophisticated approaches related to genome level histocompatibility or using kernels such as polygenic risk scores (PRS) for HSCT. In the genome era of today, the value of screening a small number of candidate polymorphisms seems low, although it may be justified if functionally relevant variants are identified that are expected to affect the outcome of HSCT. However, without genome level analysis even positive findings may merely reflect linkage disequilibrium, as opposed to genuine causative variation.

Machine learning for genomic data and the integration of genomic data with other omics data are a highly active area of statistical and bioinformatic research in the field of complex diseases, either for modeling, i.e. understanding the disease, or for prediction. Gene-set analyses might be particularly important. Here, the association of a whole SNP set with an outcome (14) is tested, thus reducing high dimensionality and allowing for biological insights. Selecting genes, pathways or regions for such tests yields possible post-GWAS analyses, even with current available sample sizes. For many complex diseases, e.g. psychosis, the implication of immunological pathways have been shown this way (15). Machine learning tools, such as kernel methods, analyze which part of the variation in the outcome can be explained globally by a non-parametric unknown function, based on the SNP set after adjusting for other variables, e.g. clinical variables, possibly in the usual parametric way. The function does not need to be specified directly, but its impact on variation can be tested *via* the so-called kernel which measures similarity between individuals (16).

The PRS as an additive sum is a particular, extremely simple kernel which is computationally fast. However, there are much more sophisticated kernels available which, e.g. allow for interactions within pathways (17). It should be noted that the similarity kernel above describes the similarity or dissimilarity between two patients, e.g. with respect to their PRS, and not the similarity between the recipient-donor pair. Thus, for cGvHD this additional similarity needs to be integrated into the model as well. Machine learning tools such as boosting or shrinkage can be used in order to extract the most important features. If features are defined by gene-sets, boosting might be combined with kernel approaches, as in Friedrich et al. (18) for rheumatoid arthritis, which is heavily influenced by the HLA locus. This overwhelming effect might make it challenging to detect other signals without the use of selection approaches that take this into account – similar to the approach needed in cGvHD. In addition to these purely genomic analyses, genomic data might be integrated with other omics data, either by enrichment with external information, such as the gene-expression data base (gtex), or when other omics data are available for the same patients and donors. For the latter, several kernels might be combined in one analysis, thus e.g. assessing similarity on the genomic as well as the transcriptomic level. Most of the research on a consolidated modeling approach has been performed on GWAS-transcription analysis, however, these are quickly extended to other omics data. For prediction, feature selection on several omics levels is computationally more feasible. However, validation is the key issue for the development of prediction tools for all complex diseases, as for cGvHD or outcome of HSCT in general. Due to the pairing of recipient and donor, the dimensionality regarding several omics level is increased, so that feature selection is expected to have a larger influence in HSCT. Some of the mentioned approaches may also need extensions in order to deal with the competing risks in HSCT. The number of GWAS of HSCT is increasing, but the globally available number of recipient-donor pairs to study is clearly lower than many other diseases. Thus, these new methods using dimensionality reduction and the increasing number of available GWASs enable the use of the modern approaches for these patients.

## Genetic Polymorphisms Associated With cGvHD

There is an excellent paper by Martin et al. (11), who searched for SNPs reported to be associated with cGvHD prior to August 2014, and then tested which SNPs could be replicated in two independent cohorts of HSCT. The two cohorts had sufficient numbers of cases, 4000 and 3200 cases, for meaningful conclusions to be drawn. The original studies had substantially lower cohort numbers of HSCTs, and were mostly based on very limited candidate gene approaches in retrospective materials.

Martin and co-workers performed a PubMed search using the terms “chronic GvHD” and “polymorphisms” and identified 29 candidate SNPs potentially associated with cGvHD. Only 5 of the 21 SNPs tested, those located near or in the *CTLA4*, *HSPE*, *IL1R1*, *CCR6*, and *FGFR1OP* genes, could be replicated in a study setting identical to that described in the original report. As the study by Martin et al. included large cohorts, these five genes, may be regarded as promising genetic biomarkers for cGvHD. However, the few GWAS analyses on GvHD conducted to date, although mostly focussed on aGvHD, have not found supporting evidence for any of these five genes (11, 12, 19). Larger GWAS studies are clearly needed to resolve the discrepancy.

To identify more recent studies we performed a PubMed search identical to that of Martin et al. (20) but focussing on post-August 2014 and identified 7 papers regarded as relevant to the present review. The associations are summarized in **Table 2**; it is of note that the majority of new studies did not focus on the markers that were confirmed by Martin et al. (20). Supporting evidence for *IL1R1* was reported by Kim et al., 2014 (27). On the other hand, no support for *IL1R* or *CTLA4* genes was identified by Hyvärinen et al. (23). Two new studies (21, 22) both report association of *IFNG* with cGvHD, but no evidence for this locus was found by Hyvärinen et al. (23). The majority of studies have reported or focused on a small number of candidate loci or SNPs, and it is not clear whether negative findings were reported.

**Table 2:** Results of PubMed search using the same terms as Martin et al., i.e., “chronic GVHD” and “polymorphisms,” only publications appearing after August 2014 have been listed.

Publication (Reference)	Gene	Population	Genome	Graft	N	SNP	Allele/genotype^a^	Statistic	95% CI	P
Martínez-Laperche et al. (21)	*IL1A*	Spanish	D	MRD	207 pairs	rs1800587	TT	OR 3.70	1.00–13.68	0.031
	*IL1B*		D			rs1143627	CT/CC	OR 0.53	0.29–0.95	0.03
	*IL1B*		D			rs16944	GG	OR 1.82	1.01–3.23	0.045
	*IL1B*		D			rs1143634	TT<CT<CC	OR 1.62	1.00–2.62	0.045
	*IL23R*		R			rs6687620	TT	OR 6.82	0.8–58.00	0.026
	*IL23R*		R			rs11209026	AG	OR 2.48	1.03–5.95	0.034
	*IFNγ*		R			rs2069705	CC	OR 1.17	0.48–2.82	0.042
Kamel et al. (22)	*INFγ*	Egyptian	R	MRD	106 pairs	rs2430561	T/T	OR 8	1.59–40.20	0.012
Hyvärinen et al. (23)	*IL1B*	Finnish	R	MRD	239 Finnish pairs	rs16944	A	OR 1.55	1.00–2.38	0.047
	*NOD2*	Finnish	R			rs6500328	G	OR 1.58	1.03–2.41	0.035
	*HSPA1L*	Finnish	D			rs2075800	T	OR 0.62	0.38–0.99	0.046
	*KRAS*	Finnish	D			rs1137282	G	OR 0.46	0.24–0.88	0.017
	*IL23R*	Spanish	R		253 Spanish pairs	rs11209026	A	OR 2.61	1.13–6.04	0.02
	*TLR9*	Spanish	D			rs352140	C	OR 0.58	0.37–0.91	0.018
	*TLR9*	Spanish	D			rs352139	T	OR 0.59	0.38–0.93	0.023
Dukat-Mazurek et al. (24)	IL-10- promoter	EUR	R	MRD, MUD	MRD: 70 recipients, 64 donors; MUD: 38 recipients, 17 donors	rs1800896, rs1800871, rs1800872	ATA/ATA	OR 19.5	1.0–362	0.04
	TGF-beta codon 10 T/C codon 25 C/G		R			rs1800470, rs1800471	T/T G/G	OR 2.3	1.0–5.4	0.04
	TGF-beta codon 10 T/C codon 25 C/G		D			rs1800470, rs1800471	T/T G/G	OR 6.5	1.2–34.0	0.02
Koyama et al. (25)	*REG3A*	Japanese	R	MRD	126 recipients	rs7588571	non-GG	OR 2.6	1.1–6.0	0.029
Berro et al. (26)	*TGF-β1 +29*	Argentinean	D	MRD	245 pairs	rs1800470	CC	HR 9.0	1.3–62.0	0.02
Kim et al. (27)	*IL1R1*	Canadian	D	MRD, MMD, MUD	394 pairs	rs3917225	AG/GG	HR 1.3	1.1–1.53	0.002
	*FCGR2A*		D			rs1801274	TC/CC	HR 1.26	1.07–1.5	0.007

CI, confidence interval; D, donor; EUR, European; HR, hazard ratio; MMD, mismatched donor; MRD, matched related donor, MUD, matched unrelated donor; OR, odds ratio; P, P-value; R, recipient.

^a^The effective allele or genotype.

We also performed a broader literature search in the PubMed database, using the terms: “chronic”, “chronic GvHD”, “graft”, “host”, “biomarker”, “gene”, “SNP”, “polymorphism”, “non-HLA”, “genetic risk” and “genetic variant”, and starting from year 2000 to February 2019, in order to identify any additional studies that were previously overlooked (**Supplementary Figure 1**). In total, we found 674 papers of which 50 were regarded as relevant to the present review. We excluded reviews, seminar papers, acute GvHD only papers, non-genetic papers, papers that analyzed pediatrics only and those that reported poor statistical evidence (p > 0.05). When we further eliminated those already identified in **Table 2**, 20 additional publications remained. Their findings have been summarized in **Table 3**. Briefly, there were in total 21 different loci suggested to show association with cGvHD but of these, only recipient *IL6* rs1800795 SNP was replicated in at least one additional independent population (42, 44–46).

**Table 3:** 20 publications found in addition to those of Table 2, using broader PubMed search terms (**Supplementary Figure 1**). Only those found relevant to the present chronic GvHD review have been listed.

Publication	Gene	Population	Genome	Graft	N	Variation	Allele/genotype^a^	Statistic	95% CI	P
Norden et al. (28)	*GR*	EUR	D	MRD	458 pairs	rs6198, rs33388, rs33389	non-ACT	OR 0.374	0.16–0.85	0.025
Kielsen et al. (29)	*IL-7Rα*	EUR	D	MRD, MUD, URD	460 donors	rs6897932	T	HR 2.0	1.1–3.6	0.025
Takahashi et al. (30)	*NLRP3*	Japanese	D	MUD	677 pairs	rs10925027	T x HLA-C MM	SHR 2.02	1.30–3.13	0.002
Inamoto et al. (31)	*BANK1*	US	D	MRD, MUD, MMRD, MMUD	847 recipients, 808 donors	rs10516487	TT	HR 0.43	0.21–0.87	0.02
	*CD247*		D			rs2056626	GG or GT	HR 1.23	1.00–1.51	0.04
	*CD247*		R			rs2056626	GG or GT	HR 1.24	1.01–1.52	0.04
	*HLA-DPA1*		D			rs987870	CC or CT	HR 2.50	1.22–5.11	0.01
	*HLA-DPA1*		R			rs987870	CC or CT	HR 2.13	1.00–4.54	0.05
Resende et al. (32)	*IL17A*	Brazilian	R	MRD, MUD, MMUD	34 pairs	rs2275913	AA	NA	NA	0.03^b^
Guillem et al. (33)	*TYMP*	EUR	D	MRD	448 pairs	rs112723255	GG	HR 1.7	1.00–2.70	0.039
Shamim et al. (34)	*IL-7Rα*	Caucasian	D	URD	590 pairs	rs14944558	TT	NA	NA	0.002^c^
			D			rs1494555	GG	NA	NA	0.004^c^
Clark et al. (35)	*BAFF*	European-American	R	MRD, MUD	156 recipients, 153 donors	rs16972217	A	OR 2.72	1.38–5.39	0.004
			R			rs7993590	T	OR 2.35	1.22–4.55	0.011
			R			rs12428930	C	OR 2.53	1.27–5.04	0.008
			R			rs2893321	G	OR 2.48	1.25–4.92	0.009
Takami et al. (36)	*FCGR3A*	Japanese	R	MUD	99 pairs	rs396991	T	HR 0.45	0.20–0.99	0.049
Sellami et al. (37)	*CTLA4*	Tunisian	D	MRD	112 pairs	*rs*231775	G	OR 2.58	1.05–6.32	0.032^c^
Inamoto et al. (38)	*CCR9*	Japanese	D	MRD	167 donors	rs12721497	AG	HR 4.1	1.1–15	0.036
Kornblit et al. (39)	*HMGB1*	EUR	D	MRD, MMRD, MUD, MMUD	275 recipients, 274 donors	rs2249825	G	HR 1.53	1.15–2.05	0.004^d^
	*HMGB1*		D			rs3742305	C	HR 1.57	1.18–2.09	0.002^d^
	*HMGB1*		D			rs41376448	insT	HR 1.57	1.17–2.12	0.003^d^
Boukouaci et al. (40)	*MICA*	EUR	R	MRD	211 pairs	rs1051792	GG	HR 1.61	1.08–2.40	0.019
Bertinetto et al. (41)	*TNFA*	EUR	R	MRD	77 pairs	rs361525	GG	NA	NA	0.027^c^
Laguila Visentainer et al. (42)	*IL6*	Brazilian	R	MRD	118 pairs	rs1800795	CC	RR 3.87	1.01–14.86	0.049
	*IL6*		D			rs1800795	CC	RR 6.64	2.26–19.54	<0.001
Bogunia-Kubik et al. (43)	*IFNγ*	EUR	R	RD, URD	160 recipients	Microsatellite (CA)	3/3 genotype	OR 3.18	1.22–8.27	0.018
Stark et al. (44)	*TNFR2*	EUR	D	MRD	104 pairs	rs1061622	GG	OR 11	NA	0.024
Cullup et al. (45)	*IL1α*	EUR	D	MRD	98 patients, 94 donors	rs1800587	T	0R 4.364	NA	0.032^e^
	*IL1α*		D			Intron 6 VNTR	Allele 2	OR 6.832	NA	0.008
	*IL6*		R			rs1800795	GG	OR 5.603	NA	0.016
Cavet et al. (46)	*IL6*	EUR	R	MRD	80 pairs	rs1800795	GG	OR 4.25	1.49–12.16	0.007
Takahashi et al. (47)	*IL10*	Japanese	D^f^	MRD + URD	54 recipients	–1,064 microsatellite, CA repeats	Allele ≥13	OR 4.5	1.3–16.1	0.020

CI, confidence interval; D, donor; EUR, European; HR, hazard ratio; MMRD, mismatched related donor; MMUD, mismatched unrelated donor; MRD, matched related donor, MUD, matched unrelated donor; NA, not available; OR, odds ratio; R, recipient, RD, related donor; RR, risk ratio; SHR, subdistribution hazard ratio; URD, unrelated donor; US, United States; VNTR, variable number tandem repeat.

^a^The effective allele or genotype.

^b^Chi-square test, frequencies only.

^c^Univariate analysis, not significant in multivariate analysis.

^d^Results significant only in the myeloablative cohort, non-significant in the nonmyeloablative cohort.

^e^Not significant if donor VNTR is also included.

^f^“Donor-derived”, DNA samples are from recipients after the transplantation.

Based on this analysis, it is clear that there are currently no adequately-proved single genetic biomarkers for cGvHD. It also became apparent that we should no longer focus on just a small number of candidate genes or SNPs in small study populations. Sato-Otsubo et al. (11), Bari et al. (12), Goyal et al. (19) and Hyvärinen et al. (23) have published GWASs on GvHD, however, the results are diverse and have no major focus on the chronic form of GvHD. Hence, larger collaborative GWAS are needed. Another fruitful approach would be to study the entire linkage disequilibrium blocks covering each of the best candidate genes as a whole, considering all markers, genotyped on an array or imputed, in order to elucidate whether the suggested associations remain valid and to identify the primary-associated variation.

The standard candidate gene or SNP approach, as well as GWAS, essentially look for associations of a single gene variant or polymorphism with the trait in question. For example the occurrence of cGvHD after allo-HSCT. Studies on other complex traits have strongly indicated that it is very rare to find a single locus with a strong effect, rather, a high number of common variants each with a small effect as well as a considerable number of rare variants can be assumed. In immunological diseases, the role of HLA variation as a susceptibility factor seems to be the only example of a strong single gene or locus effect (48), whereas the effect sizes of all other susceptibility loci are individually very small. Recent evidence by Khera et al. (49) suggests that it is possible to utilize GWAS data so that once a sufficient number of cases have been analyzed, genome-wide polygenic risk scores (PRSs) can be calculated based on associated markers. The PRS for one outcome is an additive sum of many SNPs, with the lowest p-values in a large GWAS weighted according to their effect size. In a (possibly much) smaller secondary study, interest may lie in how much variation this PRS explains or how well it predicts the same or a related outcome, e.g. identifying magnitude of risk for individuals and thus identifying low, medium, and high risk categories, Khera et al. (49) could identify highest risk individuals, such as for coronary artery disease (CAD), with surprisingly good accuracy by improving standard PRS construction with a Bayesian approach and subsequent shrinkage based on local linkage disequilibrium. In this study the number cases for CAD was >60.000 and the number of controls even higher. There have been no attempts to test PRS for GvHD so far, as crucial GWAS data for PRS construction are missing, but in the future, building of larger consortia, improvements on PRS construction as well as GWAS data on the related autoimmune diseases as primary GWAS may enable this approach as a powerful novel tool to real life practice.

There are some recent interesting attempts toward using a PRS-type of summary effect of associated markers, as well as more sophisticated data analysis methods to create genetic summary risk scores for GvHD. Martinez-Laperche and co-workers (21) built a predictive model based on clinical variables and genetic variation in a set of cytokine genes. The authors analyzed the data using a linear regression model and Lasso approach in 509 HSCTs. The risk scores, albeit not actual PRSs, could identify both severe acute GvHD and extensive cGvHD with good accuracy. The area under the curve in the receiver operating characteristic analysis of the model combining both the clinical and genetic data was 0.8 for extensive cGvHD. Secondly, Ritari et al. utilized machine learning tools to genome-wide polymorphism data to predict for complications of HSCT and reported promising results for predicting relapse (7). For cGvHD, the approach gave more modest results. Finally, Balavarca and co-workers (50) refined the EBMT clinical risk score for survival prediction by adding results of polymorphisms in the MyD88-adapter like, estrogen receptor, IL-10 and IL-6 genes in a cohort of 762 HSCT from 7 European transplantation centers.

## Associations With Polymorphisms Located Within MHC Segment

There is evidence for an increased risk of cGvHD in patients having an HLA-matched unrelated donor, compared to those with an HLA-identical sibling donor (51, 52). As the determination of HLA alleles can be assumed to be reliable using today’s DNA-based methods, this risk may not therefore derive from hidden allelic differences in unrelated HSCTs. Rather, it could indicate that the MHC region contains alleles or polymorphism associated with cGvHD, in addition to the established role of HLA allele matching in GvHD risk. It is also likely that the higher overall genomic difference between unrelated individuals, as compared to siblings, can explain the higher risk for cGvHD as we later discuss.

The HLA or MHC gene segment is one of the densest genomic regions and encompasses at least 269 genes in ~3.8 Mbp of DNA (53, 54). It is associated with more than 100 diseases (48, 53), but not all of these associations are directly caused by variants of classical HLA class I or II genes, given the linkage disequilibrium within this region and the unexpectedly high polymorphic diversity of other genes encoded in the MHC complex (55). Therefore, MHC-linked, non-HLA genetic variation is an interesting focus area for GvHD-related risk markers. Petersdorf and co-workers screened over 1000 MHC-region SNPs for their role in HSCT-related complications in a total of over 5000 HSCTs (56, 57). Genetic associations between MHC-linked SNPs were found for e.g. mortality, relapse and acute GvHD, but for cGvHD only a matching effect could be seen (see section “Histocompatibility or matching of genetic variation as predictive biomarker for cGvHD”). In the GWAS performed on Finnish and Spanish allo-HSCT, including over 5000 SNPs in the MHC region, no evidence for genetic association between cGvHD and MHC-linked SNPs was found at the genome-wide level (58). Based on these studies and that of Ritari et al. (59) it is likely that the genetic risk to cGvHD may be more related to overall genomic matching, as opposed to associations with single markers.

However, a number of candidate gene approaches indicate that the MHC segment may contain some gene variation associated with cGvHD and we discuss the most promising of these below. In the case of *TNFA* and the MHC class I chain-related molecules A gene *(MICA)*, we have some understanding of possible mechanisms underlying the association with cGvHD. In our literature search, we identified seven retrospective studies describing an association between MHC-linked non-HLA variants and cGvHD (21, 23, 40, 41, 60, 61). Four of these studies report associations between *TNFA* variation with cGvHD (21, 41, 60, 61), one reports an association with *MICA* (40), and another indicates an association with the heat shock protein family A (*HSP70*) member 1 like gene (*HSPA1L*) (23). As the MHC segment has a strong linkage disequilibrium (LD), it is very difficult to pinpoint the genuine causative gene for the associations and we cannot rule out that some, or even all, of these findings stem from a single variant. It is also possible that the genes have combinatorial effects.

### Effects of TNFA

Mullighan and colleagues studied 22 polymorphisms in 11 candidate genes in a cohort of 160 patients undergoing HSCT, based in three Australian transplant centers between 1991 and 1998 (60). 154 patients had sibling donors and 6 received grafts from other relatives. Of three SNPs in the *TNFA* gene that were analyzed (+488, -238, and -308), the *TNFA* +488 A allele in recipients showed association with an increased risk of cGvHD (odds ratio [OR] 12.5, 95% confidence interval [CI] 1.6–99.3, P = 0.003). Unfortunately only a univariate analysis was performed, leaving the genuine value of the result unknown (60).

A single center study from Italy evaluated 15 non-HLA, MHC-linked polymorphisms including seven SNPs in the *TNFA* gene (-863, rs1800896; -857, rs1799724; -574, -376, rs1800750; -308, rs1800629; -244, rs673; -238, rs361525) in a cohort of only 77 patients transplanted with grafts from HLA-identical sibling donors (41). Presence of the *TNFA* -238 A allele was found to be associated with a lower risk of cGvHD (P = 0.0027).

In a cohort from a single center in Brazil, including 122 patients transplanted between 1996 and 2006 with grafts from HLA-identical siblings, one candidate SNP in the *TNFA* gene and 2 *IL2* SNPs were analyzed (61). The GA genotype of recipients in the *TNFA* -238 SNP was found to be associated with increased incidence (risk ratio [RR] 2.04, 95% CI 1.04–4.04, P = 0.0039) and severity (RR 2.59, 95% CI 1.29–5.19, P = 0.0074) of cGvHD.

A more recent study by Martinez-Laperche et al. (21), analyzed 25 candidate SNPs in 12 genes encoding cytokines in a cohort of 509 Spanish patients who received HLA-identical sibling allo-HSCT. Four SNPs in the *TNFA* gene were studied: rs1800629 (-308), rs1800610, rs361525 (-238), and rs1799964 (-1031). None of them were associated with cGvHD in the initial univariate analysis, however, all were included in the prediction model for occurrence of cGvHD (see above).

The three first *TNFA* studies above were disadvantaged by a small number of patients and a univariate approach. As they furthermore report contradictory results, the validity of these findings remain open. In particular, systematic studies (21, 23, 62) with larger cohorts and marker sets failed to find associations of *TNFA* polymorphisms with cGvHD. Nonetheless, among the non-HLA candidate genes that have been evaluated for association with aGvHD, *TNFA* SNPs are among the few that have shown some consistency in their associations with outcome in different settings of HSCT. The linkage disequilibrium of the *TNFA* polymorphisms with HLA-haplotypes leaves room for effects by other genes. Since occurrence of aGvHD is one of the most important clinical risk factors for cGvHD, it is possible that *TNFA* polymorphisms secondarily affect the risk of cGvHD by modulating the risk of aGvHD, by controlling TNF-α levels during the cytokine storm following HSCT. However, an association of functionally relevant *TNFA* gene variants with the outcome of HSCT could be biologically plausible. Increased serum levels of TNF-α after HSCT have been associated with an increased risk or increased severity of cGvHD in several, albeit not all, reported studies as reviewed recently by others (63). The rs1799964 (-1031), rs1800629 (-308), and rs361525 (−238) SNPs in the promoter of *TNFA* have been described in multiple studies (64–75) to affect the expression level of this cytokine, suggesting a potential mechanism by which these SNPs could modulate the risk of cGvHD.

### Effects of MICA

*MICA* is the most polymorphic non-classical HLA class I-like gene (http://www.ebi.ac.uk/imgt/hla/). MICA is constitutively expressed in only a few tissues, such as gastrointestinal epithelium (76), but it can be induced by cellular or genotoxic stress (76–78). MICA is a ligand for the activating receptor NKG2D, expressed by most natural killer (NK) cells and CD8^+^ T cells (79). Proteolytic shedding can generate soluble MICA (sMICA), which can induce NKG2D down-regulation resulting in impairment of both NK cell cytotoxicity and activation of CD8^+^ T cells (79).

SNP rs1051792 at nucleotide position 454 (G/A) of *MICA* causes a valine (Val) to methionine (Met) exchange at amino acid position 129, which separates *MICA* alleles into two groups. *MICA* variants containing a methionine at position 129 bind NKG2D with high affinity, whereas those with a valine bind with low affinity (80, 81). The potential importance of this variation is highlighted by numerous disease associations including GvHD (81) that have been reported for this SNP (82).

Boukouaci and colleagues studied the MICA-129 SNP in a cohort of 211 patients who underwent non-T cell-depleted HSCT between 1994 and 2002 in a single center in France (40). In a multivariate analysis, the recipient MICA-129Val/Val genotype was associated with an increased risk of cGvHD (hazard ratio [HR] 1.61, 95% CI 1.08–2.40, P = 0.019). Other independent risk factors for cGvHD were aGvHD, older age and peripheral blood as the source of stem cells. Notably, the MICA-129Met/Met genotype in patients was associated with an increased risk of relapse (HR 1.91, 95% CI 1.02–3.58, P = 0.04). Moreover, elevated levels of sMICA in sera of patients post transplantation were associated with an increased incidence of cGvHD, whereas the presence of anti-MICA antibodies conferred protection against cGvHD. However, it needs to be mentioned that another recent study from the USA including 552 patients with unrelated donors failed to show any association between the MICA-129 SNP in patients and the outcome of HSCT (83).

The association of high-affinity MICA-129Met variants with an increased risk of relapse and low avidity MICA-129Val variants with an increased risk of cGvHD (40) appears to be counterintuitive, but it might be biologically feasible. Functional studies (81) revealed that the MICA-129Met isoform triggers increased NK cell cytotoxicity and IFN-γ release than the MICA-129Val isoform. Similarly, the MICA-129Met isoform induced an earlier co-stimulatory activation of CD8^+^ T cells than the MICA-129Val isoform. However, when the expression intensity of MICA is taken into consideration, the biological effects of this SNP can change. The extent of functional responses of NK cells and CD8^+^ T cells were found to correlate closely with the MICA expression intensity only for the MICA-129Val isoform. Increasing the expression of the MICA-129Met isoform, in contrast, had either none or even negative effects on the activation of NK and CD8^+^ T cells. This is most likely because high affinity MICA-129Met ligands induced a faster and stronger down-regulation of NKG2D than MICA-129Val ligands. The down-regulation of NKG2D impairs then the capability of NK and CD8^+^ T cells to receive signals *via* NKG2D. Thus, MICA-129Met ligands elicit strong NKG2D responses but stimulate in parallel a robust negative feedback signal. Extensive down-regulation of NKG2D appears to limit the initially stronger effects of MICA-129Met ligands. Carriers of the MICA-129Val/Val genotype might therefore be less able to limit the activation of alloreactive CD8^+^ T cells, resulting in an increased risk of cGvHD. Carriers of the MICA-129Met/Met genotype, on the other hand, might have lower CD8^+^ T and NK cell responses against malignant cells resulting in an increased risk of relapse. Recently, a well-powered study indicated that patients having a donor carrying MICA-129Met have a lower risk of grade 3 and 4 aGvHD and non-relapse mortality (84). However, the high relation of donor and recipient *MICA* genotypes is a challenge for ascribing the effects to donor or patient genotypes.

The SNP MICA-129 is an instructive example of how complex the functional consequences of even a single polymorphism can become when taking into consideration the effects on different cells, effects depending on expression intensity, and effects depending on the timing of expression. Notably, the SNP itself also appears to affect MICA expression. The MICA-129Met isoform was found to be retained longer in intracellular compartments and when transported to the cell surface, it was more prone to shedding than the MICA-129Val isoform (85). Both processes could limit expression of the high affinity MICA-129Met isoform at the cell surface. Effects of sMICA and occurrence of anti-MICA antibodies obviously further complicate the situation (40).

### Effects of *HSPA1L*

*HSPA1L* (*HSP70-HOM*) is another non-HLA MHC-linked gene that has recently been associated with cGvHD (23). Hyvärinen and colleagues analyzed 122 SNPs that have been previously described as GvHD-associated in two cohorts receiving grafts from HLA-matched siblings, in Finland (n = 301) and in Spain (n = 264). In the Finnish cohort, the donor T allele of SNP rs2075800 in the *HSPA1L* (*HSP70-HOM*) gene was found to be associated with protection from cGvHD (OR 0.62, 95% CI 0.38–0.99, P = 0.046). SNP rs2075800, which leads to a Glu-Lys amino acid change at position 602 of the protein, has been studied previously for an association with aGvHD in Poland (86) and the UK (87). The most convincing evidence for the role of HSPA1L in HSCT comes from Petersdorf et al., who reported in their screen of over 5000 allo-HSCT a protective effect against aGvHD for the rs2075800 AA and AG genotypes, both with the hazard ratio of ~0.7 (62).

*HSPA1L* is one of three *HSP70* genes encoded in the MHC class III region (88). The other two genes *HSPA1A* (*HSP70-1*) and *HSPA1B* (*HSP70-2*) encode an identical protein, *i.e.* the major heat-stress inducible HSP70. The heat stress-inducible HSP70 is a molecular chaperone (89) that has in addition to its functions in protein folding, manifold effects in the immune response (90, 91). However, *HSPA1L* is constitutively expressed at a reasonable level only in the testis and is not heat-stress inducible (92), arguing against direct involvement in GvHD. The three orthologous genes in the rat and mouse MHC show the same expression pattern. Hyvärinen and colleagues reported results from the blood expression quantitative trait loci (eQTL) database (93), showing that the protective donor rs2075800 minor allele T was associated with increased expression of *HSPA1A* and *HSPA1B* (23). It is of note that the coding sequence of HSPA1A is within the *HSPA1L* gene, but in the opposite strand, and the *HSPA1L* gene is located very closely to the *HSPA1B* gene. Again, these results cannot pinpoint the variation primarily associated with GvHD.

A direct involvement of the stress-inducible HSP70 in the pathophysiology of GvHD is more plausible than a role for the testis-specific HSP70 isoform. In rat models, expression of HSP70 was induced in spleen and lymph nodes during aGvHD (94) and anti-HSP70 antibodies were found in the serum (95). Anti-HSP70 antibodies were also reported to occur in the serum of patients with aGvHD (96). Moreover,upregulation of HSP70 during an acute GvH reaction has been observed in human skin explant assays, in which recipient skin is co-cultured with donor lymphocytes (97). In rat skin explant assays, mRNA expression of the stress-inducible *Hspa1a* and *Hspa1b* genes, but not the *Hspa1l* gene, correlated with the grade (I to IV) of the acute GvH reaction (98). In human skin biopsies, *HSPA1L* mRNA expression was lower in patients with severe aGvHD (grades II–III) when compared to those with no or low grade aGvHD (grades 0–I) and normal controls (99). In blood, however, it was upregulated in patients with cGvHD and *HSPA1B* mRNA expression was also higher in patients with both aGvHD and cGvHD (99). The HSP70 protein, in contrast, was not found in the serum of patients with aGvHD (100). Notably, the inhibition of HSP70 has been recently suggested as a new treatment option for aGvHD. Inhibition of HSP70 appears to decrease the number of intermediate monocytes, which are major producers of pro-inflammatory cytokines during aGvHD (101).

The non-HLA genes in the MHC segment are of great interest for the pathophysiology of cGvHD, because many of the 160+ protein coding genes in this region are directly involved in the immune response (48, 53). However, the linkage disequilibrium with the classical HLA class I and class II genes and other variation in the MHC makes it very challenging to identify causative variants in the MHC region. In HLA-identical sibling transplantations, all MHC genes are assumed to be matched between patients and donors, whereas this is not the case in HLA-matched unrelated donor transplants, adding a further layer of complexity to association studies. Three genes in this region, *TNFA*, *MICA*, and *HSPA1L* have been associated with cGvHD in one or more candidate gene studies. While it is biologically plausible that the associated SNPs have direct effects on cGvHD, *via* modulating expression of the pro-inflammatory cytokine TNF-α, or the affinity of MICA to the activating NK receptor NKG2D, it is more likely that that the SNP in *HSPA1L* acts indirectly, e.g. *via* modulating expression of the neighboring stress-inducible HSP70 genes. SNPs in *TNFA*, *MICA*, and *HSPA1L* have also been associated with the risk of aGvHD. While this further increases the plausibility for involvement of these genes in the pathophysiology of GvHD, it could alternatively suggest that associations with cGvHD are mainly secondary, since occurrence of aGvHD is a strong risk factor for cGvHD. However, some studies, e.g. on the association of MICA-129 with cGvHD (40), adjusted for this effect in the analysis. Larger collaborative and preferably prospective unbiased studies are clearly needed to confirm or reject MHC-linked risk factors for cGvHD. Experimental data on functional consequences of the genetic variants might help to understand why outcome associations after HSCT are variable in different cohorts (81) and provide information on the specific circumstances under with genetic information could be important to guide clinical decisions.

## Histocompatibility or Matching of Genetic Variation as a Predictive Biomarker for cGvHD

Compared to genetic analyses of other multifactorial traits or diseases, transplantation has a special feature related to the fact that the outcome of transplantation is a summary of two individuals, usually affected by the level of immunogenetic donor-recipient matching. Studies therefore require analysis of both donors and recipients and their combinatory effect. Matching of alleles of classical HLA genes is the golden standard in many transplantations, the exact rules depending on the organ or cells transplanted. It should be noted, however, that haploidentical HSCTs, i.e. those with only one of the HLA haplotypes matched between donor and recipient, are becoming more common. How these change the role of non-HLA matching or associations of other genes is currently unknown. In HSCT, at least three levels of donor-recipient matching can be identified:

1. Matching of HLA alleles, and established testing performed prior to HSCT;
2. Matching of established miHAs known to elicit T-cell response;
3. Matching of other genomic variation, including the killer-cell immunoglobulin-like receptor (KIR) and deletion matching, and whole genome level matching

### Matching of HLA Alleles

The MHC region spans approximately 3.8 Mb of the short arm of chromosome 6 at 6p21.3 and includes more than 269 genes (48, 53, 54). Among them are the classical HLA class I (*HLA-A, -B, -C*) and class II *(-DR*, *-DQ*, and *-DP*) genes as well as non-classical histocompatibility genes coding for e.g. HLA-E, HLA-G or MICA molecules. In addition, the MHC complex harbors a number of genes with immunological functions (48), making it an immunological ‘superlocus’ of utmost interest.

While mismatching classical HLA alleles is a strong risk factor for the development of aGvHD, it appears to have only a limited effect on the risk of cGvHD (102, 103). In a large international study, less than an 8/8 match in the *HLA-A*, -*B*, -*C*, and -*DRB1* loci was not associated with an increased risk of cGvHD (102). In a similarly large cohort from Japan by Morishima et al. (104), who studied 7898 unrelated T-cell replete HSCT, only mismatching of *HLA-C* was found to be associated with an increased risk of cGvHD (103). The authors pointed out the significance of potential differences between donor and recipients in the NK-cell receptor repertoire that should also be considered while studying the effect of *HLA-C* mismatching. Therefore, further analysis of *HLA-C* mismatches and KIR ligand/receptor combinations should help to elucidate the mechanism of *HLA-C* and KIR-related immunologic reactions and their effect on transplant outcome. Arrieta-Bolaños et al. in an *in silico* study showed association of *HLA-DPB1* mismatches with cGvHD (105).

There is increasing evidence that not all allelic differences in classical HLA genes are equal, but some may be permissive. This topic has recently been reviewed by e.g. Fleischhauer and Shaw (106) and will not be summarized further here. A novel view on HLA-matching has emerged from studies on expression differences between HLA alleles. Differences in surface expression of HLA molecules have been associated with SNPs located in the 5’ or 3’ untranslated regions (UTRs) of the genes. These effects have been documented at least for *HLA-A* (107, 108), *HLA-C* (109, 110), and *HLA-DP* (111, 112). For *HLA-C*, for example, it has been shown that a SNP in the 3’ UTR abrogates the binding of hsa-miR-148 microRNA, which allows HLA-C alleles with this SNP to escape from post-translational control, resulting in a higher cell surface expression (113). The expression model is based on the assumption that alloreactive donor T cells could more efficiently recognize patient-specific HLA alleles with high levels of cell surface expression than those with low levels.

Petersdorf et al. (110) examined the role of HLA-C expression levels in *HLA-C*-mismatched HSCT. They found that the expression levels were associated with unfavorable outcome. However, no relationship with cGvHD incidence was found. Furthermore, expression differences in HLA-DP molecules, due to rs9277534 SNP (496A/G) variation in 3’ UTR of the gene, were analyzed in relation to GvHD by Petersdorf et al. (112). This polymorphism was not only associated with HLA-DPB1 expression, but was also reported to be associated with an increased risk of aGvHD. No association with cGvHD was reported. In summary, it is currently unclear whether expression differences between HLA alleles play a significant role in cGvHD susceptibility.

### Established mHA

At least 20 mHAs have been identified to date (9). Their identification is based on studies showing immunogenetic differences in mHA-encoding genes between allo-HSCT pairs, leading to a T-cell alloresponse against the difference. The role of matching mHA in GVHD susceptibility in general is not clear, as concluded in a large collaborative study by Spierings et al. in 2013 (9). However, mismatching of mHAs may be useful for directing GvL effect toward e.g. cells of hematopoietic origin (114, 115). The mHAs identified so far can be regarded as special cases of concept of whole genome histocompatibility, encompassing all immunogenic amino acid differences between allo-HSCT pairs, as discussed in the next sub-chapter.

### Matching of Other Variation

Genome-wide histocompatibility concept is based on the idea that any SNP that changes an amino acid sequence can be regarded as a potential minor histocompatibility antigen, leading to a GvH reaction if mismatched in a donor/patient pair and presented to the immune system by HLA molecules of antigen presenting cells. Hence, instead of studying only the known minor antigens, it can be hypothesized that in HLA-identical cases where the effect of HLA-mismatch is removed, the risk of GvHD could increase along with the increase in exonic, nonsynonymous SNP differences within each donor/patient pair. In a more refined model, the fact that a particular HLA allele preferentially binds and presents to the immune system at only certain preferred peptides (peptide-binding motifs), can be taken into account by predicting *in silico* which amino acid changes can be assumed to be relevant in each donor/patient pair. Tissue expression can also be included. It is possible, therefore, to test whether the occurrence of GvHD is associated with the overall level of mismatches in the exomes and in particular, with those differences that are immunologically relevant, i.e., peptides able to bind the patient/donor HLA molecules and peptides expressed in relevant tissues. Ritari et al. (59) estimated, based on exome sequencing of HSCT pairs, that each pair differed on average by 28 000 nine-mer peptides. They were all theoretically, but most likely not all in practice, able to elicit an alloimmune response. The result indicates a high number of potential protein-level mismatches in each transplantation.

We have identified three different types of approaches addressing general, non-HLA-matching:

1. Matching non-HLA MHC-linked variation
2. Matching candidate genes, including effect of deletion mismatching
3. Whole genome matching

#### Matching Non-HLA MHC-Linked Variation

As the MHC complex includes a high number of genes many with immunological functions, and as many autoimmune diseases show associations with MHC, it is possible to assume that the effect of MHC in transplantation may be more complex than merely the classical HLA matching. A small number of recent studies support this hypothesis; however, many of these studies are restricted by a very limited number of variants studied. Due to strong LD in MHC, it is impossible to obtain reliable results by focusing on only a few SNPs, instead more systematic screening is required.

Petersdorf et al. (62) screened over 1000 SNPs located in the MHC for their role in HSCT. They tested the hypothesis that clinical outcome of HSCT depends on the cumulative effects of MHC-linked SNP mismatching. Twelve SNPs were found to be significantly associated with the outcome of HLA-matched unrelated HSCT. The risks associated with these SNPs were significant in a multiple regression analysis and were conferred by either donor or recipient SNP genotype or by donor-recipient SNP mismatch. Patient-donor mismatching at rs2523957, located 26.6 kb centromeric to *HLA-A*, rs2071479 located within the intron of *HLA-DOB*, and rs3830076, located 240 bp telomeric to *FKBPL*, were found to be independent risk factors for cGvHD, regardless of grades III–IV aGvHD. Thus, the authors concluded that prospective patient-donor matching for these three SNPs may help to lower the risk of cGvHD that occurs independently of aGvHD symptoms. It is of interest that the three MHC-linked SNPs are located quite far from each other, suggesting e.g. haplotypic differences or multiple factors.

In the same study, Petersdorf et al. analyzed whether patient-donor HLA-mismatches were associated with specific SNPs. Polymorphism in SNP rs3830076, located 240 bp telomeric to *FKBPL*, differed along with the two most frequent *HLA-C*-mismatches, C*03:03 vs. C*03:04 and C*01:02 vs. C*02:02. In patients with chronic GvHD, 58.8% of C*03:03/03:04 mismatches had a protective rs3830076 variant, in contrast to 26.1% of C*01:02/02:02 mismatches. These observations suggest that C*03:03 vs. C*03:04 mismatch might be more readily tolerated. This type of information could be further used to select HLA-mismatched donors, which are less likely to result in cGvHD after transplantation.

A similar approach was conducted by Nowak et al. (116), who hypothesized that the increased risk of post-transplant complications might be dependent on disparity in non-routinely-tested polymorphisms in the MHC region, being organized in combinations of two extended MHC haplotypes. They tested the hypothesis that clinical outcome in unrelated HSCT with a certain level of HLA-mismatch is affected by the level of HLA-inferred extended MHC haplotype disparity. In their study, the overall incidence of cGvHD and extended cGvHD were found to be significantly affected by haplotypic disparity level and remained independent prognostic factors in a multiple regression analysis, in which HLA-mismatch levels were excluded from models.

Due to recombination hotspots that are located in the MHC segment, in particular between the *HLA-DQB1* and *-DPB1* genes (117), matching of *HLA-A*, *-C*, *-B*, *-DRB1*, *-DQB1* alleles only will not necessarily implicate full MHC matching. Indeed, even within families up to 5% of otherwise 10/10-matched siblings will be *HLA-DPB1*-mismatched (118). Koskela et al. (119) performed detailed genome-level analyses of HLA-matched siblings and showed that the number of hidden mismatching within MHC is more common than assumed. In most cases, the mismatching included not only the known mismatch, but many other additional genes. Furthermore, the authors found evidence that the higher level of mismatching in MHC was associated with an increased risk for both acute and chronic GvHD.

Bogunia-Kubik and co-workers found that patient-donor matching for *HLA-E* alleles might be of prognostic value for the transplant outcome and reduce the risk of aGvHD in 10/10 HLA-matched transplants (120, 121). However, this association has not been observed in a more recent study (122) and no association with cGvHD was found. HLA-E is an interesting candidate, as it influences both the innate and the adaptive arms of the immune system by engaging inhibitory and activating receptors on NK cells and CD8^+^ T cells (123). Like classical HLA class I molecules, HLA-E is ubiquitously expressed. However, it is characterized by only a very limited sequence variability, and two dominant functional alleles differing at one amino acid position in the α2 heavy chain domain have been reported. These isoforms vary in peptide binding affinity. In its role in both NK and CD8^+^ T cell regulation HLA-E resembles that of MICA.

Mismatches in *MICA* gene, located close to *HLA-B*, increased complications of HSCT in two large studies that, unfortunately, were limited to screening of *MICA* only (124, 125). Fuerst and colleagues (124) evaluated 2172 HSCT cases and found that HSCT with a 10/10 HLA-match but a MICA position 129 mismatch resulted in lower overall and disease-free survival and an increased risk of aGvHD. Carapito and co-workers (125) studied 922 French HSCT and concluded that mismatch in *MICA* alleles increased, among others, the risk of cGvHD with a HR of 1.45–1.55. It is currently unclear how *MICA* mismatching could confer such profound biological effects, but it might involve an educational tuning of NKG2D responses in NK cells to the presence of high or low affinity ligands in an individual (126).

#### Matching Candidate Genes, Including Deletion Mismatching

A number of reports on candidate non-MHC genes associated with occurrence of GvHD have also tested whether their matching between donor and recipient showed association with GvHD. Most studies are limited by too low numbers of patients and in many cases, it has been difficult to understand possible mechanisms behind the matching effect, hence, we do not go into details in this review. One potentially interesting concept was introduced by McCarroll and co-workers, who studied whether mismatched homozygous gene deletions are associated with GvHD (127). When the donor has a homozygous deletion of protein coding gene, thus missing the protein, it can be assumed that donor immune cells detect the protein as foreign, leading to a GvHD reaction. Indeed, the study screened 1345 HLA-identical HSCT and an association with aGvHD was found. No results from cGvHD were reported. It would be of interest to analyze the summary effect of all homozygous deletions between HSCT pairs. The role of copy-number variation has also been indicated in kidney transplantation (128).

#### Whole Genome Matching

Intuitively, the genome-wide histocompatibility concept, in which a summary of all immunogenetic mismatches in the genomes of donor and recipient is taken into account, could be more relevant in autoimmune-like cGvHD compared to its relevance in aGvHD. In aGvHD, the strong cytokine storm may lead to extensive immune activation that overruns the effect of other factors. The concept of genome wide histocompatibility in cGvHD has been tested in a few settings. Martin et al. (129) studied full HLA-matched sibling and unrelated HSCTs and *HLA-DP*-mismatched unrelated HSCTs for genome-wide minor histocompatibility effects. They found no evidence of an increased risk for cGvHD when genome-wide mismatching was increased, instead they concluded that the higher risk for GvHD in unrelated HSCT was due to mismatching of *HLA-DP* or other HLA genes. These results strongly pointed to the primary role of HLA-matching. On the other hand, Ritari et al. (59) assessed the exome sequences of 157 HSCT pairs and after various modeling techniques, found evidence that among HLA-identical sibling HSCTs a higher number of genome-wide mismatches predisposed patients to cGvHD. The group of Claude Perreault (130) introduced proteogenomic-based screening of overall mismatching in HSCT and utilized the findings to identify novel cell-therapy targets. Supporting the long-term effect of overall mismatch levels in transplantation, Mesnard et al. (131) reported that in kidney transplantation long-term graft function, rather than acute rejection, was associated with their genome-wide matching score. These studies provide interesting starting points for further analyses, however, at the present time the field is not mature enough for strong conclusions.

## Effect of NK Cell KIR Receptors

The role of KIR receptors is an excellent example of the complexity of biomarkers in HSCT. Their effect is influenced by various factors such as graft source, HLA matching, overall transplantation setting and appear donor or recipient specific. We therefore review the major findings of applying KIR genotypes in predicting for occurrence of cGvHD after HSCT. Many excellent and detailed reviews of KIR genetics and NK cells have been published during the past 5–10 years (132).

The function of NK cells is controlled by various surface receptors, including type I integral proteins with immunoglobulin like domains (KIR), type II integral proteins with lectin-like domains (NKG2D, CD94/NKG2) and natural cytotoxicity receptors (NCR) (133–136). Most studies to date have focused on inhibitory and activating KIRs. KIR haplotypes can be divided into two groups; A and B, depending on their KIR gene content (137). KIR A haplotypes are conserved, containing up to seven genes, most of which encode inhibitory receptors, while KIR B haplotypes have greater variation in gene content, including additional activating genes. KIR molecules recognize polymorphic epitopes at amino acid positions 76–83 of HLA class I molecules. C1 and C2 epitopes of HLA-C are recognized by KIR2DL2/2DL3 and 2DL1/2DS1, respectively. The Bw4 epitope is recognized by KIR3DL1 and the A3/11 epitope is recognized by KIR3DL2. The KIR gene region is located on chromosome 19q13.4, hence, segregating independently from HLA. During recent years, many studies have addressed the association of KIR receptors and their matching in HSCT.

### Association of KIR Gene Variation With cGvHD

A number of studies have indicated that the donor KIR gene composition, in particular the B haplotype, influences the outcome of HSCT at least in patients with acute myeloid leukemia (AML). However, whether the effect is seen in cGvHD is not clear. The results of associations of cGvHD with single KIR genes are heterogeneous.

In an analysis of patients with AML transplanted from HLA-matched (N = 209) and HLA-mismatched (N = 239) unrelated donors, Cooley et al. (138) showed that overall and relapse free survival were significantly higher in HSCT from a donor with at least one copy of the KIR B haplotype. In this study, the KIR B haplotype was associated with a higher incidence of cGvHD that may be linked to the low relapse rate. In another study, Cooley et al. (139) demonstrated that the centromeric genes in the KIR B haplotype of the donor had a strong effect on improving outcome of HSCT in AML patients. They were associated with a decreased incidence of relapse and improved disease free survival, but showed no significant effect on acute or chronic GvHD. No associations with KIR haplotypes were observed in patients with acute lymphocytic leukemia (ALL). Bachanova et al. (140) analyzed 573 HSCT recipients with chronic lymphocytic leukemia and did not find any effect of donor KIR genotype on transplant outcome. In a study of 281 patients with AML and ALL transplanted from matched sibling or unrelated donors, Faridi et al. (141) observed no effect of KIR B haplotype of the donor.

A study by Venstrom et al. (142) showed that the donor *KIR3DS1* genotype had a beneficial effect in HSCT in a cohort of 1087 patients. The incidence of aGvHD, overall mortality and transplant-related mortality decreased with an increase in the number of *KIR3DS1* copies in the donor, but there was no association with cGvHD. Giebel et al. (143) studied 100 patients with hematological malignancies transplanted from HLA-matched or single-mismatched donor and showed that the presence of activating KIR genes in the donor and their absence in the recipient was associated with a higher incidence of cGvHD. In a multiple regression analysis, aGvHD was associated with *KIR2DS1* and cGvHD with *KIR2DS3*. Zhao et al. (144) analyzed 65 patients with haploidentical HSCT receiving grafts without T-cell depletion and observed that donor KIR2DS3 was also associated with a higher incidence of acute and chronic GvHD. In contrast, the presence of donor KIR2DS3 was a protective factor for cGvHD in a study of McQueen et al. (132) based on HLA-matched T cell-repleted HSCT from sibling donors. Kamenaric et al. (145) analyzed the impact of alleles of *KIR2DS4*, the only activating KIR gene in haplotype A, on transplantation outcomes and showed that donor full-length *KIR2DS4* alleles were associated with a lower overall survival rate, a higher risk of GvHD and relapse.

### Association of KIR Matching With cGvHD

The role of NK alloreactivity and KIR HLA matching in HSCT was introduced by Ruggeri et al. (146). They showed that in haploidentical T-cell depleted HSCT from related donors with KIR ligand incompatibility to graft-versus-host direction, NK cell alloreactivity was associated with a reduced occurrence of relapse and GvHD. Alloreactive NK cells were associated with GvL effect and could prevent GvHD by killing recipient`s antigen presenting cells that initiate GvHD (146). A recent study in 144 patients with T cell–replete haploidentical HSCT with post-transplant cyclophosphamide (147) supported earlier observations. Symons et al. (148) showed in 86 patients that mismatches for inhibitory KIR between the donor and recipient were associated with lower relapse and improved overall survival. However, no significant differences regarding the incidence of acute or chronic GvHD were observed.

Other studies showed variable results. Shimoni et al. (149) in a study of 444 patients with AML and ALL after T cell-replete haploidentical transplants with cyclophosphamide showed no significant effect of KIR ligand mismatching on transplant outcome and acute or chronic GvHD. Zhao et al. (142) analyzed 65 patients with haploidentical HSCT receiving grafts without T-cell depletion and observed an increased incidence of cGvHD in patients with only one KIR ligand compared to patients with 2 or 3 KIR ligand groups, based on the number of KIR ligands in recipients (HLA-C1, HLA-C2, or HLA-Bw4).

Analysis of 178 patients (150) transplanted from HLA-identical siblings showed that in patients with AML, lack of ligands for donor inhibitory KIRs was associated with a significantly lower relapse rate, but the risk of GvHD was not different. In a small cohort of HLA-matched sibling HSCT, Wang et al. (151) observed a lower incidence of cGvHD in C1 or C2 homozygotes than in C1/C2 heterozygotes. Thus, HLA-KIR-mismatch was associated with a lower incidence of cGvHD. In a recent study, Sahin et al. (152) analyzed the relevance of KIR gene-gene matching in 96 patients with myeloid malignancies receiving HSCT form HLA-identical siblings. cGvHD occurred less frequently in case of activating or inhibitory KIR matching. The donors positive for the KIR B haplotype were associated with an increased incidence of cGvHD.

Similarly to haploidentical allo-HSCT, Giebel et al. (153) demonstrated that KIR ligand mismatching is associated with a better outcome after HSCT from unrelated donors. KIR ligand mismatches were associated with a higher overall survival and disease free survival. The majority of studies (154–159) have, however, reported adverse effects of KIR ligand mismatching in HSCT from unrelated donors. Unfortunately, only a few of these studies addressed cGvHD, hence, the results are not conclusive. Morishima et al. (156) showed that KIR ligand mismatch in the graft-versus-host direction, defined when the donor`s KIR 2DL epitope on HLA-C was not shared by patient epitope, was associated with an increased rate of aGvHD, but had no effect on the incidence of cGvHD. In another large multicenter study including 1,571 patients with myeloid malignancies transplanted from unrelated donor, Farag et al. (160) did not show significant association of KIR ligand mismatching with acute or chronic GvHD. In a study of 142 leukemic patients receiving HSCT and ATG *in vivo* T-cell depletion, Kröger et al. (161) showed no associations of KIR ligand mismatch, KIR genotypes or KIR haplotypes with chronic or acute GvHD.

In a study of 281 patients with AML and ALL transplanted from matched sibling or unrelated donors, Faridi et al. (141) found an increased incidence of cGvHD in KIR genotype mismatching between the donor and recipient when the recipients had one or more C1 bearing HLA-C epitopes.

In summary, many studies over the last two decades have focused on the role of KIR receptors and their HLA class I ligands in HSCT. The results, however, are heterogeneous and no general conclusions can be made, in particular as only some studies reported association with cGvHD. In haploidentical HSCT, decrease of cGvHD rate was associated with donor KIR B haplotype, while the opposite association was observed in other types of HSCTs. Presence of donor activating *KIR 2DS3* gene was found as a risk factor for an increase of cGvHD in many HSCT settings. The KIR associations were observed mainly in patients with myeloid diseases.

## Pharmacogenetics

Postgraft immunosuppression is an essential component of allogeneic HSCT, with the primary goal to ensure engraftment and prevent the development of GvHD. Importantly, immunosuppressants are also used to treat GvHD. The most commonly used immunosuppressive agents are calcineurin inhibitors cyclosporine and tacrolimus, dihydrofolate reductase inhibitor methotrexate, and purine metabolism inhibitor mycophenolate mofetil. Genetic polymorphisms may impact the pharmacokinetics, i.e. absorption, distribution, metabolism and elimination of these agents (162, 163).

To date, the majority of GvHD-related pharmacogenetic findings have focused on the effects on aGvHD (10) and e.g. methylenetetrahydrofolate reductase and thymidylate synthase genotypes have been associated with aGvHD in recipients receiving methotrexate (164). In 2015, a review article by Franca et al. (165) summarized studies on pharmacogenetics variants and GvHD in both adult and pediatric cohorts. Despite the observed genotype effects on pharmacokinetic parameters, the studies found no statistically significant effects on cGvHD. In 2018, McCune et al. (166) studied 247 recipient-donor pairs and discovered that in recipient genotype, mycophenolate mofetil metabolism-related inosine monophosphate dehydrogenase type 1 rs2278293 was associated with a reduced risk for cGvHD.

The role of pharmacogenetics in a cGvHD setting is largely unknown and adequately powered pharmacokinetic and pharmacodynamic studies are challenging to complete, due to the substantial heterogeneity in the conditioning regimen, graft type, postgraft immunosuppression, and clinical classifications. However, variants with large effect sizes should be detected in well-powered GWASs. The blood level of immunosuppressants could be targeted and phenotype and meta-analysis approaches may also provide more reliable results. Better understanding of the role pharmacogenetics of immunosuppressants in the initiation and development of cGvHD could be translated to clinics and be of benefit to HSCT patients. Altogether, this important field requires further studies.

## Conclusions

The present review screened scientific publications on genetic biomarkers for predicting development of chronic GvHD, a complex, autoimmune complication of HSCT. We modeled three different models for genetic factors: genetic association, genetic matching and pharmacogenetics. The review demonstrates that currently, there are no genetic polymorphisms or genetic tools that are validated, reliable biomarkers for predicting cGvHD.

Current lessons from genetic analyses of other multifactorial diseases suggest that larger, genome-wide studies with well-characterized cohorts are needed. The majority of the studies on HSCT thus far have included too few patients and have focused on only a small number of markers. Some candidate genes, such as *CTLA4*, *HSPE*, *IL1R1*, *CCR6*, *FGFR1OP*, and *IL10*, and HLA-linked MICA have been replicated to be associated with cGvHD risk in independent studies, and replications of these findings with fine mapping arrays or sequencing in various populations are needed.

A notable limitation of studies on cGvHD is the fact that clinical criteria of cGvHD changed over time and that they might not always have been interpreted consistently between centers. Moreover, progressive quiescent and *de novo* cGvHD, which are usually not distinguished in the studies, could have significant pathophysiological differences. This heterogeneity could also contribute to the inconsistency of results.

It is of note that a number of patients develop cGvHD in spite of very extensive immunosuppression and other treatments. Perhaps the medication is sufficiently effective in patients with specific genome profiles. Only sporadic publications on pharmacogenetic studies in cGvHD were found and this key area clear needs urgent attention. It should be noted, however, that GWAS also address variation in the drug metabolism genes. However, in this regard they are hampered by the variety of treatments in addition to the variety of underlying diseases.

Another line of research that may yield important findings is the use of modern genomic tools for cGvHD. Machine learning for genomic data and the integration of genomic data with other omics data could be employed for modeling and prediction. In addition to summarized effects of PRSs, other modern kernels may enable interactions within pathways. Genomic data might be integrated with other omics data from public gene-expression data bases or from the same individuals. The increased dimensionality induced by recipient-donor pairing could be tackled by machine learning tools selecting important features.

Finally, the risk of developing cGvHD may not be related to any single biomarker, but to the summary level of immunogenetic differences between each donor-recipient pair, that is, to the whole genome histocompatibility level. There is some evidence for this in cGvHD (59, 130) and also in long-term outcome of kidney transplantation (131). The differences may also include gene-deletion mismatches. Intuitively, the whole genome histocompatibility could influence the risk of chronic rather than aGvHD, in which the whole genome histocompatibility differences may be overruled by the cytokine storm and consequent immune activation.

## Data Availability Statement

All datasets presented in this study are included in the article/**Supplementary Material**.

## Author Contributions

RC, KH, and FP made the literature searches and compiled their results. RC revised the language in the revision. KB-K drafted the HLA sections and a part of *Introduction* section. MI drafted the KIR section. RD drafted the non-HLA MHC section and a part of *Introduction* section. JP drafted *Introduction, Summary, Conclusions* and the whole genome studies sections. KH drafted the pharmacogenetics section and a part of *Introduction* section. HB drafted parts on statistical/bioinformatics methods in the revision. JP, KH, and RD finalized the manuscript. All authors contributed to the article and approved the submitted version.

## Funding

The authors are supported by the following grants: JP and KH are supported by the Academy of Finland, the Finnish Cancer Fund, the SalWe “Get it done”-project from the Tekes (currently Business Finland), and the VTR funding from the government of Finland. KB-K is supported by a grant from the National Science Centre (Poland): 2018/31/B/NZ2/03065. RC is supported by the Newcastle upon Tyne Hospitals NHS Charity. RD has been supported by the Deutsche Forschungsgemeinschaft (GRK 1034) and the European Union grant FP7-PEOPLE-2012-ITN-315963 (CELLEUROPE). MI is supported by grant NSF (Bulgaria) project H23/7 (contract KP-06-H23/4). All authors are supported by the COST foundation for project CA17138 cGvHD Eurograft (https://GvHD.eu).

## Conflict of Interest

The authors declare that the research was conducted in the absence of any commercial or financial relationships that could be construed as a potential conflict of interest.

## Supplementary Material

Supplementary Figure 1 Flow chart (top) and search terms (below) of PubMed literature search for genetic biomarkers of chronic GVHD.
