# Supplementary figures and images for: Review of Genetic Variation as a Predictive Biomarker for Chronic Graft-Versus-Host-Disease After Allogeneic Stem Cell Transplantation
Source: Front Immunol. 2020 Oct 19;11:575492. doi: 10.3389/fimmu.2020.575492 (PMC7604383; doi:10.3389/fimmu.2020.575492)

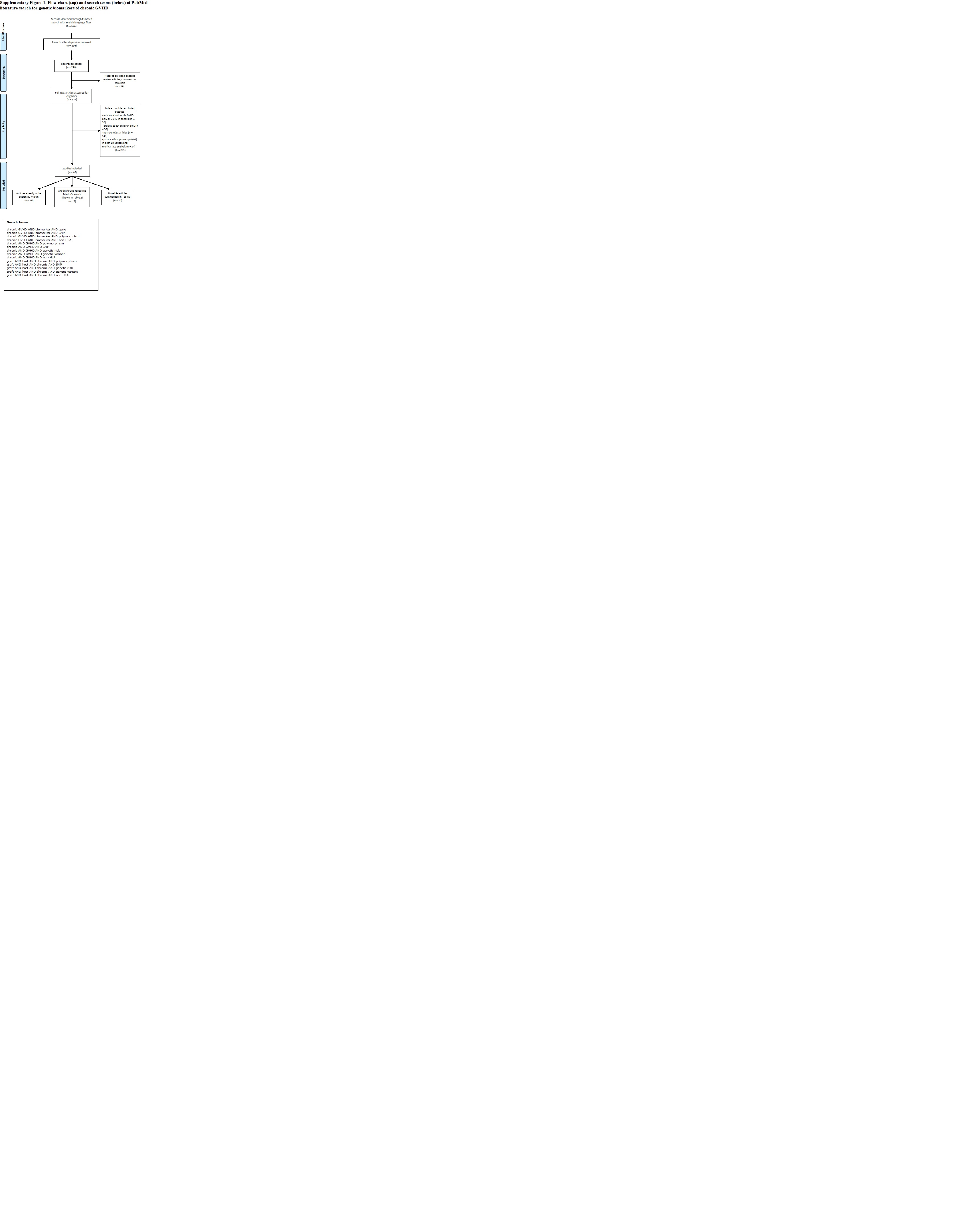

Supplement: Supplementary Figure 1 — Flow chart (top) and search terms (below) of PubMed literature search for genetic biomarkers of chronic GVHD. [file Image_1.jpg]
